# Routine *MDM2* FISH Testing and Preoperative Biopsy Reduces Recurrence Rate of Atypical Lipomatous Tumours

**DOI:** 10.1002/jso.70349

**Published:** 2026-07-29

**Authors:** Samuel O. H. Ching, Anderson S. M. Leung, Raymond C. H. Yau, Albert Y. L. Lam, Tony W. H. Shek, Maximus C. F. Yeung

**Affiliations:** ^1^ Department of Pathology, School of Clinical Medicine, LKS Faculty of Medicine, Queen Mary Hospital The University of Hong Kong Hong Kong SAR China; ^2^ Orthopaedic Oncology and Limb Salvage Surgery, Department of Orthopaedic and Traumatology Queen Mary Hospital Hong Kong SAR China

**Keywords:** ALT, atypical lipomatous tumour, FISH, MDM2, preoperative, recurrence

## Abstract

**Background:**

Although *MDM2* FISH test has been increasingly used to differentiate ALT and benign lipomatous tumours, its impact on clinical outcome remains underexplored. This study investigated whether routine *MDM2* FISH testing on preoperative biopsy is associated with a reduction in the recurrence risk of ALT.

**Methods:**

A total of 336 patients with *MDM2*‐verified well‐differentiated lipomatous tumours (ALT, *n* = 67; lipoma, *n* = 269) treated at a tertiary sarcoma centre were retrospectively analysed. Clinicopathological parameters, histological–molecular concordance, pre‐operative diagnostic approach and recurrence rates were compared.

**Results:**

Compared to benign lipomatous tumours, ALT patients were older (*p* = 0.002), had larger tumours (median 15.0 vs 7.0 cm, *p* < 0.001) and more frequently deep extremity lesions (*p* < 0.001). Up to 25% of biopsy specimens of ALT exhibit purely benign morphology. ALT patients who underwent preoperative biopsy with *MDM2* FISH had a lower recurrence rate than those who did not (16.2% vs 56.7%, *p* < 0.001). This was associated with a higher rate of extracapsular excision (97.3% vs 71.4%–77.8%, *p* = 0.021).

**Conclusion:**

Routine *MDM2* FISH testing on preoperative biopsy provides a definitive diagnosis of ALT, especially for lipoma‐like histology, enabling surgeons to plan extracapsular excision and thereby reducing local recurrence.

## 1 Introduction

1.1

Atypical lipomatous tumour (ALT) is the most common lipomatous tumour with biological potential, being locally aggressive [1]. Typical histological features include variation in adipocytic sizes, atypical stromal cells in thickened fibrous septa featuring enlarged hyperchromatic, pleomorphic stromal cells, and the presence of lipoblasts with multivacuolation [2]. ALT commonly occurs in the proximal extremities and trunk. The term ‘well‐differentiated liposarcoma’ (WDLPS) is reserved for atypical lipomatous tumour occurring in deep‐seated sites such as retroperitoneum, mediastinum and spermatic cord, which is associated with a much higher risk of dedifferentiation and disease progression [1, 3].

ALT has a recurrence rate of approximately 15% in case of incomplete excision [3, 4], compared to approximately 5%–7% for benign lipoma [5]; it is also associated with 10% risk of dedifferentiation [3]. Differentiating ALT from lipoma is quintessential for deciding treatment approach, with extracapsular excision (marginal excision or wide excision) recommended for ALT [6]. Because of the similar clinical and radiological features between ALT and lipoma [7], pre‐operative biopsy is suggested for lipomatous tumours larger than 3–5 cm in size or deep‐seated location [8, 9]. As atypical cells may only be focally observed in ALT and subtle misdiagnosis as benign lipoma may occur, especially in small biopsy specimens [10].

On the molecular level, ALT is characterised by high‐level amplification of the Murine double minute 2 (*MDM2*) gene at 12q15 region with ring chromosome formation [11, 12], leading to inactivation of the *TP53* tumour suppressor gene and driving oncogenesis. Testing for *MDM2* amplification helps to distinguish between benign lipoma and ALT [13], especially in histologically equivocal cases [14]. *MDM2* fluorescence in situ hybridisation (FISH) represents the gold standard simple molecular test for diagnosing *MDM2* amplification, in which a positive case typically features a high copy number for the *MDM2* gene as compared to the diploid centromere of chromosome 12 [11, 12]. *MDM2* immunohistochemistry (IHC) may potentially serve as a good surrogate marker, but the overall sensitivity and specificity are only approximately 80% and 90%, respectively [15], compared to 93.5% sensitivity and 100% specificity for *MDM2* FISH [16].

*MDM2* FISH testing is routinely performed in our tertiary referral specialised sarcoma centre, as well as many other major centres in other parts of the world. While previous studies demonstrated the suboptimal histological assessment for diagnosing ALT without the availability of FISH results [17], with only a 50% concordance rate between histology and *MDM2* FISH for histologically equivocal cases [18], the clinical outcome of routine *MDM2* FISH test on preoperative biopsy is largely unexplored. In this study, we investigate whether the routine pre‐operative biopsy and *MDM2* FISH test is associated with a reduction in the recurrence risk of ALT.

## 2 Materials and Methods

### 2.1 Patient Cohort and Data Collection

Patients diagnosed with ALT and benign lipomatous tumours from 2016 to 2024 in Queen Mary Hospital (QMH), a specialised bone and soft tissue tumour tertiary referral centre in Hong Kong, were retrospectively analysed. The diagnosis of each case has been validated by the status of the *MDM2* FISH test, in which ALT were positive for *MDM2* gene amplification, whereas benign lipomatous tumours were negative for *MDM2* gene amplification. Cases without an excision specimen were excluded. The clinical data (age, sex, site, size, depth, margin, surgery type, recurrence) were extracted from the Clinical Management System (CMS) of the Hospital Authority, Hong Kong. The clinical parameters at the first diagnosis were used for analysis. The size was defined as the maximal dimension of the tumour (in centimetres) on gross examination of the pathology report or based on magnetic resonance imaging (MRI) findings. Cases of recurrence include both pathological recurrence (diagnosed by biopsy) and radiological recurrence (detected by MRI). Histological features were reviewed by expert soft tissue pathologists (M.C.F.Y. and T.W.H.S.): atypical morphology was defined as the presence of atypical stromal cells possessing hyperchromatic, pleomorphic nuclei or lipoblasts, and benign morphology referred to the absence of atypical features. The study was approved by the Institutional IRB (UW22‐168).

### 2.2 *MDM2* Fluorescent In‐Situ Hybridisation (FISH)

*MDM2* FISH test was performed using Vysis MDM2/CEP12 FISH Probe Kit (Abbott Molecular, cat no: 01N15‐010). The *MDM2* gene on chromosome 12 was targeted by the orange probe, and the centromeric region of chromosome 12 was targeted by the green probe. The targeted area on the H&E section of the tissue was marked by a pathologist, and it was viewed under a fluorescence microscope. The signal patterns for 20 tumour nuclei were counted, and nuclei with no signals or with signals of only one colour were not scored. Cells were considered negative if the number of orange signals in a cell was < 10; cells were considered positive if the number of orange signals in a cell was >= 10 or clusters of orange signals were observed. The specimen was considered *MDM2* gene not amplified if < 10 cells out of 20 (< 50%) were positive, while the sample was considered *MDM2* gene amplified if > = 10 cells out of 20 (> = 50%) were positive.

### 2.3 Statistical Analysis

The statistical analyses and Kaplan‐Meier (KM) curve were performed using Python (PyCharm 2025.3). For comparison of different clinicopathological parameters, the Chi‐square test was used for categorical variables, and the Mann‐Whitney *U* test was used for continuous variables without normal distribution. A *p*‐value of < 0.05 was considered to be significant.

## 3 Results

### 3.1 Comparison of ALT Versus Lipoma

The clinicopathological features of the current cohort were summarised in Table 1. It comprised 336 patients with *MDM2‐*verified lipomatous tumours, including 67 cases of ALT (65 lipoma‐like, 1 sclerosing, 1 inflammatory) and 269 cases of lipomas (201 typical lipoma, 41 intramuscular lipoma, 13 spindle cell lipoma, 9 fibrolipoma, 4 angiolipoma, 1 lipoma with myxoid change).

**Table 1:** Clinicopathological differences between MDM2, done ALT and lipoma.

Clinicopathological parameters	ALT (*n* = 67)	Lipoma (*n* = 269)	*p*‐value
Sex, *n* (%)			
− Men − Women	32 (47.8%) 35 (52.2%)	118 (43.9%) 151 (56.1%)	0.585
Age (years), median (range)	64.0 (39.0–92.0)	58.00 (1–88)	**0.002**
Preoperative biopsy done, *n* (%)			
− Yes − No	47 (70.1%) 20 (29.9%)	167 (62.1%) 102 (37.9%)	0.257
MDM2 FISH done before surgery, *n* (%)			
− Yes − No	37 (55.2%) 30 (44.8%)	150 (55.8%) 119 (44.2%)	1.000
Site, *n* (%)			
− Trunk − Extremities	11 (16.4%) 56 (83.6%)	101 (37.5%) 168 (62.5%)	< **0.001**
Site (subclassification), *n* (%)			
− Head and neck − Chest wall − Abdominal wall − Upper back − Lower back − Upper extremities − Lower extremities	0 (0.0%) 7 (10.4%) 0 (0.0%) 1 (1.5%) 1 (1.5%) 11 (16.4%) 47 (70.1%)	23 (8.6%) 15 (5.6%) 4 (1.5%) 45 (16.7%) 18 (6.7%) 102 (37.9%) 62 (23.0%)	< **0.001**
Size (cm), median (range)	15.0 (4.0‐33.0)	7.0 (1.0‐23.0)	< **0.001**
Depth, *n* (%)			
− Deep − Superficial	62 (93.9%) 4 (6.1%)	113 (42.0%) 156 (58.0%)	**< 0.001**
Recurrence, *n* (%)			
− Yes − No	23 (34.3%) 44 (65.7%)	7 (2.6%) 262 (97.4%)	< **0.001**

The bold values indicate the statistical significance of *p* < 0.05.

ALT and lipoma cases shared similar percentages of preoperative biopsy done (70.1% vs 62.1%; *p* = 0.257) and preoperative *MDM2* FISH performed (55.2% vs 55.8%; *p* = 1). Compared to lipomas, patients with ALT were significantly more likely to be older (median of 64 vs 58 years, *p* = 0.002), larger in size (15.0 cm vs 7.00 cm, *p* < 0.001), situated in the extremities (83.6% vs 62.5%, *p* < 0.001), and deep in location (94.6% vs 42%, *p* < 0.001). There was a significantly higher recurrence rate in ALT (34.3% vs. 2.6%, *p* < 0.001).

### 3.2 Correlation of Final Diagnosis Based on *MDM2* FISH Result and Histology

The comparison of histology with final diagnosis based on *MDM2* FISH result was summarised in Table 2. Discordance between histology in both biopsy and excisional specimens and the *MDM2* FISH result was identified. Among cases of ALT, up to 25% of biopsy specimens exhibited benign morphology, and even for excisional specimens, 9% of cases showed completely benign morphology, the so‐called lipoma‐like ALT (Figure 1A,B) and reminiscent of intramuscular lipoma (Figure 1C,D). As for the lipoma cases, more than 4% of cases display atypical morphology, and nearly 2% of cases possess atypical morphology on excisional specimen (Figure 2).

**Table 2:** Correlation of histology and MDM2 results.

	ALT (*n* = 48)	Lipoma and variants (*n* = 171)	*p*‐value
Biopsy morphology			**< 0.001**
Benign	12 (25%)	164 (95.9%)	
Atypical	36 (75%)	7 (4.1%)	
	ALT (*n* = 66)	Lipoma and variants (*n* = 269)	*p*‐value
Excision morphology			**< 0.001**
Benign	6 (9.0%)	264 (98.1%)	
Atypical	60 (90.9%)	5 (1.9%)	

The bold values indicate the statistical significance of *p* < 0.05.

**Figure 1:**
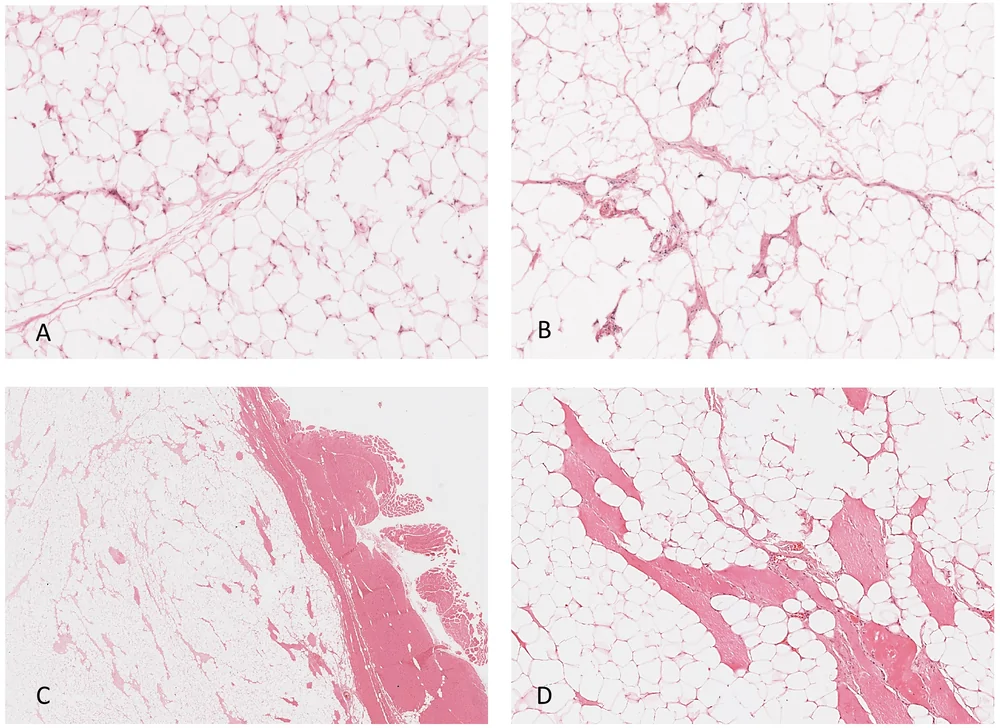
Histology of atypical lipomatous tumour (ALT) with benign morphology. (A) & (B) Lipoma‐like ALT featuring mild variation in adipocytic sizes with minimal atypia in the mildly thickened fibrous septa. (C) & (D) Histology of a case of ALT originally misdiagnosed as intramuscular lipoma leading to recurrence.

**Figure 2:**
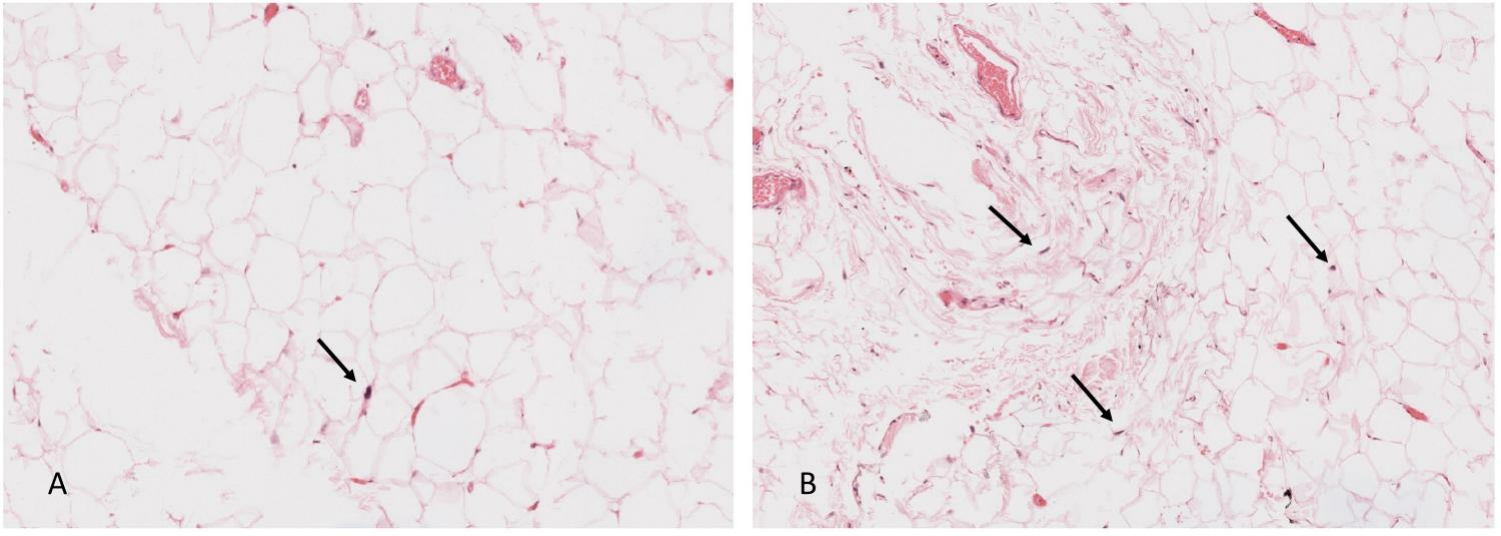
Histology of lipoma cases featuring a focal area of atypical adipocytes and stromal cells.

### 3.3 *MDM2* FISH Testing on Preoperative Biopsy Was Associated With a Reduction in the Recurrence of ALT

Next, we investigated the clinicopathological factors associated with recurrence of ALT (Table 3). Preoperative biopsy alone was associated with a reduction in the recurrence rate from 60% to 23.4% (*p* = 0.006). With *MDM2* FISH test on preoperative biopsy, it was associated with a significantly greater reduction of recurrence from 56.7% to 16.2% (*p* < 0.001). Among patients with preoperative biopsy done, adding *MDM2* test was associated with further reduction of the recurrence risk from 50% to 16.2% (*p* = 0.039). No clinicopathological parameters were associated with a significant difference in recurrence rate, including patient demographic data (age and sex) and tumour characteristics (site, size, margin status and surgery type). Patients with *MDM2* FISH test done in pre‐operative biopsy achieved the best recurrence‐free survival compared to others without preoperative biopsy or *MDM2* FISH test, though it did not reach statistical significance (Figure 3).

3.4

**Table 3:** Differences in clinicopathological factors and status of preoperative biopsy and/or MDM2 test between recurrence and non‐recurrence ALT cases.

Parameters	Recurrence (*n* = 23)	No recurrence (*n* = 44)	*p*‐value
Age (years), median (range)	59.0 (40–82)	65.0 (39–92)	0.070
Sex, *n* (%)			
− Men − Women	12 (52.2%) 11 (47.8%)	20 (45.5%) 24 (54.5%)	0.618
Site, *n* (%)			
− Trunk − Extremities	3 (13%) 20 (87%)	8 (18.2%) 36 (81.8%)	0.736
Site (subclassification), *n* (%)			
− Head and neck − Chest wall − Abdominal wall − Upper back − Lower back − Upper extremity − Lower extremity	0 (0.0%) 2 (8.7%) 0 (0.0%) 0 (0.0%) 0 (0.0%) 7 (30.4%) 14 (60.9%)	0 (0.0%) 5 (11.4%) 4 (9.1%) 1 (2.3%) 1 (2.3%) 4 (8.5%) 33 (75.0%)	0.217
Size (cm), median (range)	13.0 (4.0–33.0)	16.0 (5.0–25.5)	0.526
Margin, *n* (%)			
− R0 − R1	3 (21.4%) 11 (78.6%)	17 (43.6%) 22 (56.4%)	0.203
Surgery type, *n* (%)			
− Both + Extracapsular − Intracapsular	15 (83.3%) 3 (16.7%)	38 (90.5%) 4 (9.5%)	0.412
Preoperative biopsy done			
− Yes − No	11 (23.4%) 12 (60%)	36 (76.6%) 8 (40%)	**0.006**
MDM2 test on preoperative biopsy			
− Yes − No	6 (16.2%) 5 (50.0%)	31 (83.8%) 5 (50.0%)	**0.039**
MDM2 test on preoperative biopsy versus no MDM2 test or no biopsy			
− MDM2 test on preoperative biopsy − No MDM2 test or no biopsy	6 (16.2%) 17 (56.7%)	31 (83.8%) 13 (43.3%)	**< 0.001**

The bold values indicate the statistical significance of *p* < 0.05.

**Figure 3:**
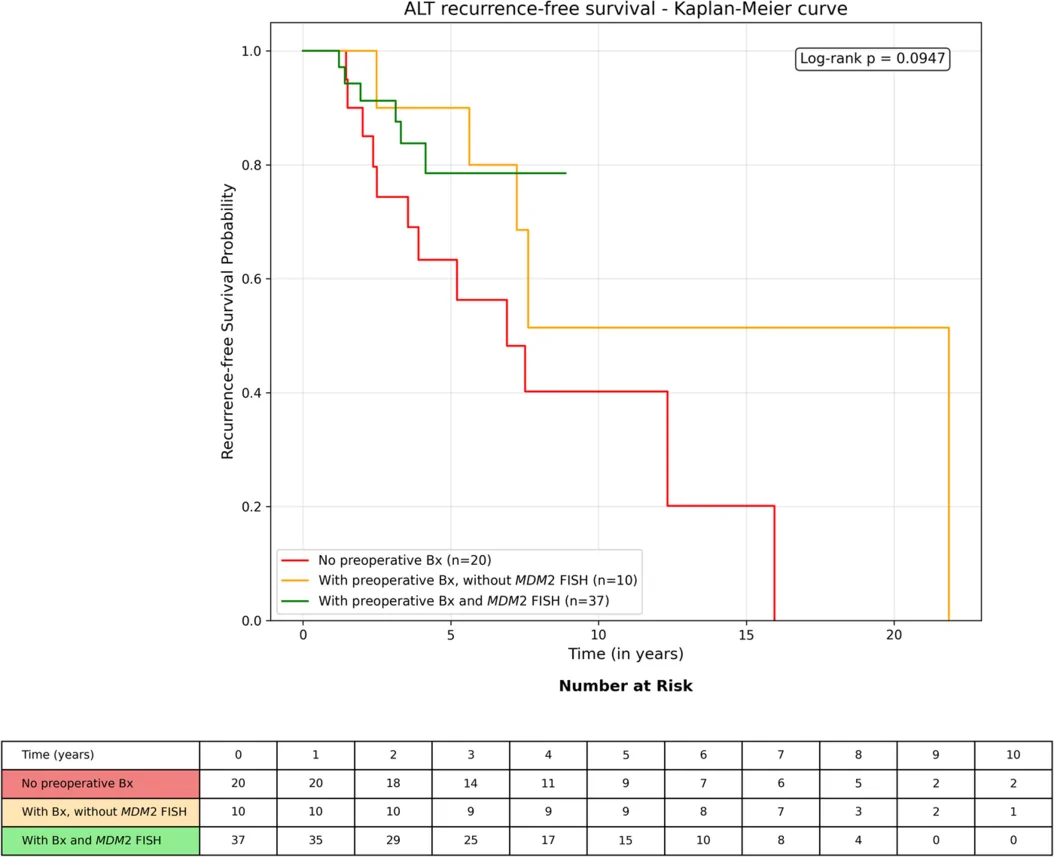
Kaplan‐Meier curve for ALT patients without pre‐operative biopsy, with preoperative biopsy without *MDM2* FISH, and with preoperative biopsy and *MDM2* FISH.

## 4 Comparison of ALT Stratified by Availability of Preoperative Biopsy and *MDM2* FISH Test

To evaluate the impact of preoperative diagnostic work‐up on surgical management and outcomes, ALT cases were stratified into three groups according to the performance of preoperative biopsy and *MDM2* FISH testing (Table 4). ALT cases with biopsy and *MDM2* FISH performed preoperatively, compared to cases with preoperative biopsy alone or without preoperative biopsy and *MDM2* FISH, were more likely to be situated in the extremities (94.6% vs 90% vs 60%, *p* = 0.003), especially the lower extremities (83.8% vs 70% vs 45%, *p* = 0.018), with larger size (17 cm vs 16 cm vs 11 cm, *p* = 0.033) and in the deep location (100% vs 100% vs 78.9%, *p* = 0.005). Most importantly, the extracapsular approach was more likely to be adopted for ALT cases diagnosed with both preoperative biopsy and *MDM2* FISH (97.3% vs 77.8% vs 71.4%, *p* = 0.021), which also showed the lowest recurrence rate (16.2% vs 50% vs 60%, *p* = 0.002).

**Table 4:** Clinicopathological differences among different preoperative diagnostic work‐up strategies.

Clinicopathological parameters	With preoperative Biopsy and *MDM2* FISH (*n* = 37)	With preoperative biopsy alone (*n* = 10)	No preoperative biopsy or *MDM2* FISH (*n* = 20)	*p*‐value
Sex, *n* (%)				
− Men − Women	18 (48.6%) 19 (51.4%)	4 (40.0%) 6 (60.0%)	10 (50.0%) 10 (50.0%)	0.864
Age (years), median (range)	66.0 (39.0–92.0)	61.0 (55.0–75.0)	59.5 (40.0–82.0)	0.293
Site, *n* (%)				
− Trunk − Extremities	2 (5.4%) 35 (94.6%)	1 (10.0%) 9 (90.0%)	8 (40.0%) 12 (60.0%)	**0.003**
Site (subclassification), *n* (%)				
− Chest wall − Upper back − Lower back − Upper extremities − Lower extremities	1 (2.7%) 0 (0.0%) 1 (2.7%) 4 (10.8%) 31 (83.8%)	0 (0.0%) 0 (0.0%) 0 (0.0%) 3 (30.0%) 7 (70.0%)	6 (30.0%) 1 (5.0%) 0 (0.0%) 4 (20.0%) 9 (45.0%)	**0.018**
Size (cm), median (range)	17.0 (6.0–25.5)	16.0 (7.0–33.0)	11.0 (4.0–24.0)	**0.033**
Depth, *n* (%)				
− Deep − Superficial	37 (100.0%) 0 (0.0%)	10 (100.0%) 0 (0.0%)	15 (78.9%) 4 (21.1%)	**0.005**
Surgery type, *n* (%)				
− Both + Extracapsular − Intracapsular	36 (97.3%) 1 (2.7%)	7 (77.8%) 2 (22.2%)	10 (71.4%) 4 (28.6%)	**0.021**
Margin status, *n* (%)				
− R0 − R1	16 (47.1%) 18 (52.9%)	3 (42.9%) 4 (57.1%)	1 (8.3%) 11 (91.7%)	0.056
Recurrence, *n* (%)				
− Yes − No	6 (16.2%) 31 (83.8%)	5 (50.0%) 5 (50.0%)	12 (60.0%) 8 (40.0%)	**0.002**

The bold values indicate the statistical significance of *p* < 0.05.

## 5 Discussion

The differences of clinicopathological parameters between ALT and lipoma cases in our cohort were in line with previous studies ‐ ALT was larger in size and more likely to be located in the intramuscular layer of extremities, specifically the lower extremity [10]. The recurrence rate of ALT was higher than that of lipoma, as expected (34.3% vs 2.6%), indicating a more aggressive clinical behaviour of ALT. Determining the nature of the lipomatous tumours preoperatively enables a higher chance of complete excision of ALT by better surgical planning (extracapsular excision or intracapsular excision in case of proximity to major neurovascular structures), which in turn effectively reduces the recurrence risk and improves patient outcome [19, 20]. Multiple strategies for diagnosing ALT for lipomatous lesions have been suggested, including incorporating clinical, radiological and histological features [6, 8, 21], but definite preoperative diagnosis of ALT relies on a molecular test for the detection of *MDM2* gene amplification, especially for the ambiguous cases [13, 14]. It was echoed by our study, which demonstrated that a considerable portion of ALT cases (25% of biopsy and 9% of excision) display purely benign histology. Conversely, a few lipoma cases displayed atypical histological features (e.g. scanty hyperchromatic stromal cells), which led to erroneous ALT diagnosis and prompted wide resection with the potential of causing functional deficit. These results demonstrated the limitation of histology‐based preoperative diagnosis of ALT.

This study illustrated the relationship between routine preoperative biopsy and *MDM2* FISH testing and recurrence risk for ALT. The recurrence risk was significantly lower for cases with preoperative biopsy, and adding the *MDM2* FISH test to the preoperative biopsy showed further correlation with lower recurrence risk. This was associated with the differences in surgical approach, in which extracapsular excision was performed in cases where ALT was confidently diagnosed preoperatively. Indeed, many of the ALT cases showing recurrence were initially misdiagnosed as benign lipomatous tumours based on histology alone. In these cases, retrospective *MDM2* FISH testing on primary tumours was found to be positive after recurrence. These findings signify the clinical significance of pre‐operative biopsy and *MDM2* test for patients with ALT.

Based on previous studies, multiple criteria are recommended prior to proceeding with *MDM2* testing for well‐differentiated lipomatous neoplasms [17, 22, 23]. These included recurrent lesions, > 5 cm lesions located in deep extremity lesions for patients over 50 years old, cases with equivocal atypia, retroperitoneal/pelvic/abdominal lesions, and in special clinical situations. Some of the ALT cases in our cohort arose from patients younger than 50 years old, and the size of the lesion could be less than 5 cm (with the smallest primary tumour measuring 4 cm in greatest dimension). Adopting the aforementioned selection criteria would miss some ALT patients, who might undergo incomplete excision with subsequent disease recurrence, leading to multiple excisions and risk of disease progression (e.g. dedifferentiation). This was exemplified by the higher proportion of intracapsular excision and higher recurrence rate for ALT cases without preoperative biopsy and *MDM2* FISH in our study. Therefore, we would advocate routine *MDM2* FISH testing for all cases of lipomatous tumours with a preoperative biopsy done. For a resource‐limited setting, our experience of routine pre‐operative biopsy and MDM2 testing may not be directly applicable. Further study in such a setting is recommended.

Apart from preoperative biopsy and *MDM2* FISH testing, no clinicopathological parameters—including surgical margin status—were significantly associated with recurrence in our ALT cohort (*p* = 0.203). Recurrent cases showed a higher proportion of positive margins (78.6% vs 56.4% in non‐recurrent cases), but this trend did not reach significance, likely due in part to uncertain or missing margin data in some patients, which reduced the effective sample size for analysis. Several recent studies have similarly reported that microscopically positive (R1) margins do not significantly increase local recurrence risk in extremity ALT compared to R0 resections, with recurrence rates often comparable (7%‐8%) across margin statuses [19, 20, 24]. This supports a function‐preserving marginal excision approach, especially when tumours lie close to critical neurovascular structures, to minimise morbidity while achieving acceptable local control. Larger multicenter studies with longer follow‐up are needed to determine whether margin status has a subtle independent effect on recurrence.

One limitation of the study was the lack of inclusion of other lipomatous tumours, including but not limited to atypical spindle cell/pleomorphic lipomatous tumour and hibernoma, which might occasionally contain scattered atypical stromal cells [1] on biopsy but were negative on *MDM2* FISH. The sample size of our cohort was still relatively small. Further research on a larger cohort and including other entities of benign lipomatous tumours will be needed.

In summary, our study shows that routine preoperative biopsy with *MDM2* FISH testing is associated with a marked reduction in recurrence risk for atypical lipomatous tumours through better surgical planning from more accurate preoperative diagnosis. The addition of *MDM2* FISH on preoperative biopsy confers further clinical benefit and should be considered in settings with adequate resources.

## Conflicts of Interest

The authors declare no conflicts of interest.

## Synopsis

Routine preoperative *MDM2* FISH testing of lipomatous tumours provides a definitive diagnosis of atypical lipomatous tumour even when histology is benign, enabling planned extracapsular excision and resulting in a significantly lower recurrence rate (16.2% vs 56.7%).

## Data Availability

The data that support the findings of this study are available on request from the corresponding author. The data are not publicly available due to privacy or ethical restrictions. The data supporting the findings of this study are available within the article.
